# Expression of concern: Impact of some pyrrolidinium ionic liquids on copper dissolution behavior in acidic environment: experimental, morphological and theoretical insights

**DOI:** 10.1039/d4ra90029d

**Published:** 2024-03-25

**Authors:** Emad E. El-Katori, Ashraf S. Abousalem

**Affiliations:** a Department of Chemistry, Faculty of Science, The New Valley University El-Kharja-72511 Egypt emad_992002@yahoo.com emad.elkatori@scinv.au.edu.eg +20927925393 +201023318210; b Quality Control Laboratory, Operations Department Jotun-11835 Egypt

## Abstract

Expression of concern for ‘Impact of some pyrrolidinium ionic liquids on copper dissolution behavior in acidic environment: experimental, morphological and theoretical insights’ by Emad E. El-Katori and Ashraf S. Abousalem, *RSC Adv.*, 2019, **9**, 20760–20777, https://doi.org/10.1039/C9RA03603B.

The Royal Society of Chemistry is publishing this expression of concern in order to alert readers that concerns have been raised regarding the reliability of the AFM images in Fig. 10A–D. An investigation is underway, and an expression of concern will continue to be associated with the article until a final outcome is reached.

Laura Fisher

19th March 2024

Executive Editor, *RSC Advances*

## Supplementary Material

